# *Candida parapsilosis* Protects Premature Intestinal Epithelial Cells from Invasion and Damage by *Candida albicans*

**DOI:** 10.3389/fped.2017.00054

**Published:** 2017-03-22

**Authors:** Sara Gonia, Linda Archambault, Margaret Shevik, Marie Altendahl, Emily Fellows, Joseph M. Bliss, Robert T. Wheeler, Cheryl A. Gale

**Affiliations:** ^1^Department of Pediatrics, University of Minnesota, Minneapolis, MN, USA; ^2^Department of Molecular and Biomedical Sciences, University of Maine, Orono, ME, USA; ^3^Department of Pediatrics, Women & Infants Hospital of Rhode Island, Warren Alpert Medical School of Brown University, Providence, RI, USA; ^4^Graduate School of Biomedical Sciences and Engineering, University of Maine, Orono, ME, USA; ^5^Department of Genetics, Cell Biology and Development, University of Minnesota, Minneapolis, MN, USA

**Keywords:** *Candida albicans*, *Candida parapsilosis*, fungal pathogenesis, premature infant, intestinal epithelium, zebrafish model system

## Abstract

*Candida* is a leading cause of late-onset sepsis in premature infants and is thought to invade the host *via* immature or damaged epithelial barriers. We previously showed that the hyphal form of *Candida albicans* invades and causes damage to premature intestinal epithelial cells (pIECs), whereas the non-hyphal *Candida parapsilosis*, also a fungal pathogen of neonates, has less invasion and damage abilities. In this study, we investigated the potential for *C. parapsilosis* to modulate pathogenic interactions of *C. albicans* with the premature intestine. While a mixed infection with two fungal pathogens may be expected to result in additive or synergistic damage to pIECs, we instead found that *C. parapsilosis* was able to protect pIECs from invasion and damage by *C. albicans*. *C. albicans*-induced pIEC damage was reduced to a similar extent by multiple different *C. parapsilosis* strains, but strains differed in their ability to inhibit *C. albicans* invasion of pIECs, with the inhibitory activity correlating with their adhesiveness for *C. albicans* and epithelial cells. *C. parapsilosis* cell-free culture fractions were also able to significantly reduce *C. albicans* adhesion and damage to pIECs. Furthermore, coadministration of *C. parapsilosis* cell-free fractions with *C. albicans* was associated with decreased infection and mortality in zebrafish. These results indicate that *C. parapsilosis* is able to reduce invasion, damage, and virulence functions of *C. albicans*. Additionally, the results with cellular and cell-free fractions of yeast cultures suggest that inhibition of pathogenic interactions between *C. albicans* and host cells by *C. parapsilosis* occurs *via* secreted molecules as well as by physical contact with the *C. parapsilosis* cell surface. We propose that non-invasive commensals can be used to inhibit virulence features of pathogens and deserve further study as a non-pharmacological strategy to protect the fragile epithelial barriers of premature infants.

## Introduction

*Candida albicans* and *Candida parapsilosis* are the leading causes of invasive fungal disease in premature infants (1), with the intestinal tract being an important site for *Candida* invasion (2–4). For example, life-threatening gastrointestinal tract diseases that occur in premature infants such as necrotizing enterocolitis and spontaneous intestinal perforation are highly associated with concurrent diagnoses of invasive candidiasis. *C. albicans* and *C. parapsilosis*, along with other fungi, are prevalent commensals of the intestinal tract of infants (5–7), with high amounts of *Candida* colonization within the intestine being correlated with an increased risk for invasive disease (8). Administration of prophylactic doses of fluconazole to infants has been shown to decrease *Candida* colonization of the intestinal tract as well as the incidence of invasive candidiasis in premature infants (9). Concern remains, however, regarding off-target effects of antimicrobial agents, in particular, the impact on the developing intestinal microbiome and longer-term health (10).

*Candida albicans* is capable of forming three primary morphologies: ovoid yeast cells, chains of elongated yeast cells known as pseudohyphae, and extremely elongated filamentous cells known as true hyphae. Most *Candida* species, including *C. parapsilosis*, exist as yeast or pseudohyphae; only *C. albicans, Candida tropicalis*, and *C. dubliniensis* have been observed to form true hyphae (11). The ability to undergo hyphal morphogenesis is associated with the ability of *C. albicans* to invade and damage various human epithelial and endothelial tissues (12, 13). In particular, our laboratory has shown that *C. albicans* hyphae, but not yeast forms, cause significant invasion and damage of premature intestinal epithelial cells (pIECs) (14, 15). Other *Candida* species that do not form hyphae, including *C. parapsilosis*, are essentially inert with respect to this epithelial cell line (14).

Mixed infections that include more than one pathogen often have additive or synergistic effects on pathogenesis or virulence features as compared to infection with a single microbe. With respect to *C. albicans* infections, coinfection of vaginal epithelial cells with *C. albicans* and *C. glabrata* has been observed to result in increased epithelial cell injury as compared to infection with either single species (16). In addition, intra-abdominal infection of *C. albicans* along with *Staphylococcus aureus* results in 100% mortality in mice, whereas the mono-microbial infections are avirulent (17). Some microbes, on the other hand, have been observed to have activities that confer protection from pathogenic features of *C. albicans* infections. For example, *Pseudomonas aeruginosa* produces phenazines that inhibit the formation of *C. albicans* biofilms *in vitro* (18), and probiotic bacteria of the *Bifidobacterium* and *Lactobacillus* genera appear to reduce *Candida* colonization in extremely premature infants, although their efficacy in reducing fungal sepsis has not yet been conclusively shown (19). Collectively, these observations support the idea that the particular behavior of a microbe toward commensalism versus pathogenesis can be influenced by the relative activity of neighboring microbes.

In this study, we tested the hypothesis that *C. parapsilosis*, the second leading cause of fungal sepsis in premature infants, modulates *C. albicans*-induced damage and invasion of pIECs *in vitro*, as well as *in vivo* using a zebrafish model of candidiasis. Together, our results add mechanistic insight into pathogenic interactions between *C. albicans* and the premature intestine and how these interactions might be prevented.

## Materials and Methods

### Fungal Growth Conditions and Preparation of Cell and Cell-Free Fractions for Assays

Yeast strains (Table 1) were propagated and maintained as described previously (20). Strains were recovered from 15% glycerol stocks stored at −80°C by plating onto Yeast Peptone Dextrose agar and incubating at 30°C overnight. Individual colonies were then suspended and grown in synthetic dextrose complete medium containing 2% glucose at 30°C overnight prior to assays being performed. Cell concentrations were determined microscopically using a hemacytometer. To obtain cell-free culture fractions, yeast cells were grown as described above, sub-cultured into H4 tissue culture medium at a concentration of 2 × 10^6^ cells/mL and grown at 30°C for 12 h. Yeast cells were pelleted by centrifugation at 13,000 rpm for 3 min. The supernatants were removed carefully using a pipet, so as not to disturb the cell pellet. Supernatants were visualized microscopically using 60× magnification in multiple random fields to ensure that no yeast cells were present.

**Table 1:** **Strains used in this study**.

Strain	Description/genotype	Source
Ca SC5314	*Candida albicans* laboratory strain	(21)
Cp 4175	*Candida parapsilosis* clinical isolate, neonate, cardiac mass	(14)
Ca A022b	*C. albicans* clinical isolate, neonate, pleural fluid	(14)
Cp LOW	*C. parapsilosis* JMB72	(22)
Cp HIGH	*C. parapsilosis* JMB77	(22)
Ca GFP	*C. albicans* SC5314 *ENO1:GFP-NAT1/ENO1*	J. Berman (U of MN)

### pIEC Culture and Maintenance

Primary premature human enterocytes (cell line H4) were cultivated and maintained using H4 growth medium and conditions as previously described (23).

### Epithelial Cell Damage (Cytotoxicity) Assay

Premature intestinal epithelial cell damage was assessed as previously described (14, 24). Briefly, pIECs were cultured at a concentration of 2 × 10^4^ cells/well and grown to approximately 80% confluence in a 96-well flat-bottomed tissue culture plate (BD Biosciences, San Jose, CA, USA). The pIECs were incubated with yeast (1 × 10^6^ *C. albicans* and/or 2 × 10^6^ *C. parapsilosis*, both in ~10 μL volume), *C. albicans* (1 × 10^6^ cells) with addition of *C. parapsilosis* cell-free culture fraction from 2 × 10^6^ cells, or *C. parapsilosis* cell-free culture fraction alone. After 8 h of incubation, the amount of lactate dehydrogenase released from injured epithelial cells was measured using the Cyto-Tox-96^®^ assay (Promega, Madison, WI, USA) per the manufacturer’s instructions. The amount of cell damage is represented as a percent of the control group for each comparison and the reported results are the averages of three independent experiments, each performed in triplicate. Statistical analyses were performed as follows. For two group comparisons, Student’s *t*-test (pairing the data by day) was used. For three or more group comparisons, a blocked ANOVA was employed to account for day-to-day variation in pIEC passage number followed by *post hoc* separation of means using Tukey’s HSD.

### Invasion Assay

*Candida albicans* penetration of pIECs was determined as described previously (14, 15, 24). In general, the yeast morphologic form is non-invasive whereas the hyphal form is capable of penetration with respect to H4 pIECs (14, 15). Hyphal cells were determined to be invading or not invading pIECs after 3 h of infection with *C. albicans*, in the presence or absence of *C. parapsilosis* or *C. parapsilosis* supernatant, using an immunocytochemical method. Briefly, non-invading fungal cells are labeled fluorescently *via* the Alexa 568 fluorophore, whereas invading fungal cells are inaccessible to the primary antibody and are not labeled. Approximately 30 fungal cells were analyzed for each experimental condition and day. Statistical analysis was performed using Student’s *t*-test, pairing the data by day to account for pIEC passage number.

### Adhesion Assay

Fungal cell adhesion to pIECs was determined as described previously (25). Briefly, pIECs were inoculated into 96-well tissue culture microplates at a density of 2 × 10^4^ cells/well in a final volume of 100 μL and incubated overnight. The medium was aspirated, and pIECs were infected with 100 μL of 1 × 10^6^ *C. albicans* cells suspended in either H4 media or *C. parapsilosis* cell-free fractions and incubated for 3 h at 37°C with 5% CO_2_. Following incubation, the wells were washed with phosphate-buffered saline (PBS), the cells were fixed with 4% *p*-formaldehyde, and the yeast cells were stained with 0.5% crystal violet. The optical densities at 600 nm were determined using a spectrophotometer, and the results were expressed as the percent *C. albicans* adhesion inhibition as compared to 100% adhesion of *C. albicans* alone to pIECs. Results are reported as the average of three independent experiments, each with six replicates. Statistical analysis was performed using Student’s *t*-test, pairing the data by day to account for pIEC passage number.

### Zebrafish Growth, Maintenance, and Infection

All animal studies were carried out in accordance with the recommendations in the Guide for the Care and Use of Laboratory Animals of the National Institutes of Health. All animals were treated in a humane manner according to guidelines of the University of Maine IACUC as detailed in protocol number A2015-11-03. The UMaine IACUC/Ethics Committee approved this protocol. Animals were euthanized by tricaine overdose. Infected animals were monitored twice daily for signs of infection and morbid animals were euthanized. Wild-type AB zebrafish were maintained as described previously (26). Zebrafish larvae were grown in E3 medium (5.0 mM NaCl, 0.17 mM KCl, 0.33 mMCaCl, 0.33 mM MgSO4, 2 mM HEPES, pH 7) plus 0.3 μg/mL methylene blue for the first 6 h post-fertilization, then switched to E3 supplemented with 10 μg/mL 1-phenyl-2-thiourea to suppress pigmentation.

The *C. albicans* CAF2.1-dTom-NATr strain was used for all experiments in zebrafish (26–28) and was grown and prepared for infections as described previously (26). Overnight cultures were washed three times in calcium- and magnesium-free PBS and yeast cell concentrations were determined microscopically using a hemocytometer. Cell suspensions were adjusted to a concentration of 5 × 10^7^ cells/mL in 5% polyvinylpirrolidone dissolved in H4 media alone or fungal culture supernatants (described above in Section “Fungal Growth Conditions and Preparation of Cell and Cell-Free Fractions for Assays”).

Zebrafish larvae were infected with yeast by injection into the swimbladder at 4 days post-fertilization (dpf) as previously described (29). Infected fish were individually screened at 2 h post-infection (hpi) on a Zeiss Axiovision Vivatome microscope. Mock-infected and infected fish were divided randomly into two cohorts. One cohort of 10 fish was held for 4 days, with counting and removal of deceased fish each day. The second cohort of fish was rescreened at 24 hpi and infection parameters (swimbladder deflation and epithelial breaching) were recorded. Statistical analysis was performed on GraphPad Prism version 7.00 for Mac (GraphPad Software, La Jolla, CA, USA, www.graphpad.com). Survival curves were analyzed using a log-rank (Mantel–Cox) test. Fisher’s exact test, with Bonferroni correction for multiple tests, was used to detect differences in infection parameters among groups.

## Results

### pIEC Damage and Invasion by *C. albicans* Is Reduced by *C. parapsilosis*

**Figure 1:**
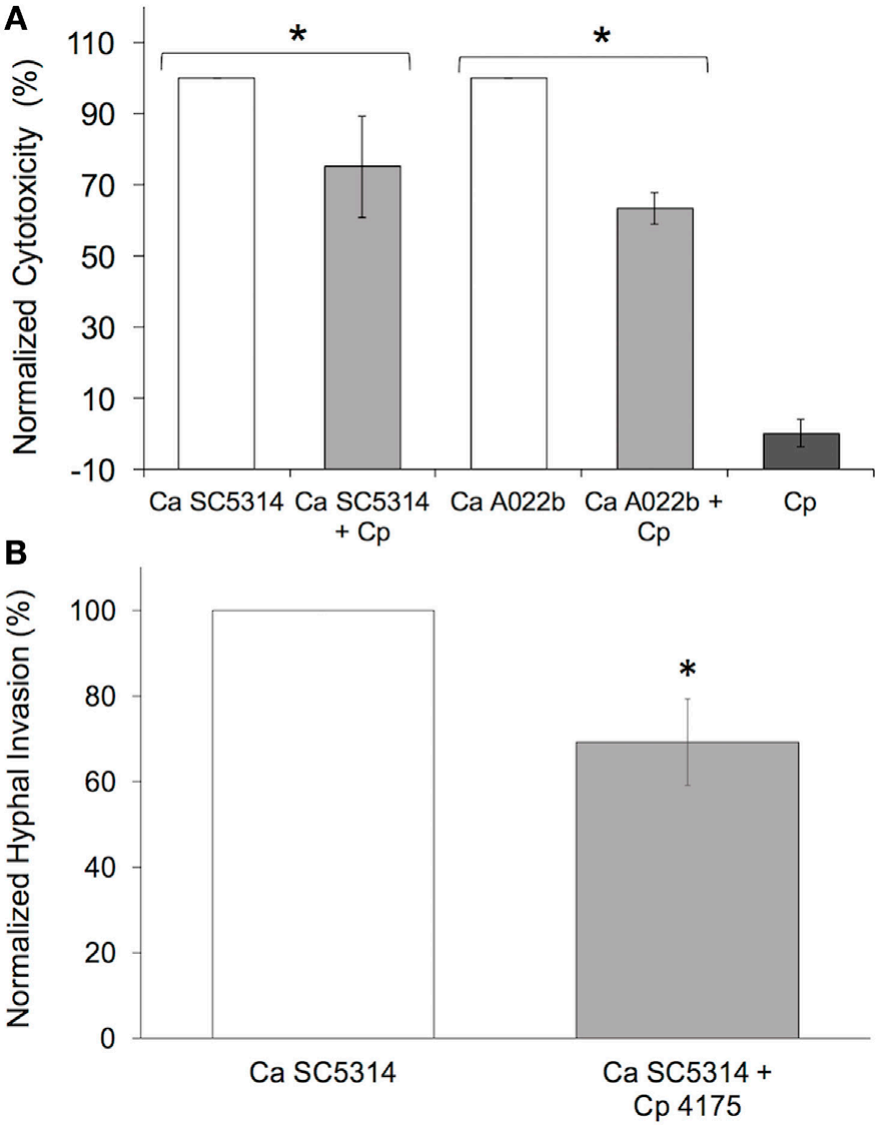
***Candida parapsilosis* reduces premature intestinal epithelial cell damage (A) and invasion (B) by *C. albicans* strains**. **(A)** Cell damage (lactate dehydrogenase amount) is plotted as a percentage of that caused by *Candida albicans* (Ca) strains (SC5314 and A022b) alone (“normalized cytotoxicity”). Cp, *C. parapsilosis* strain 4175. **(B)** *C. albicans* hyphal invasion in the presence of *C. parapsilosis* (Cp 4175) is plotted as a percentage of that observed for *C. albicans* (Ca SC5314) alone. Data shown are the average of three individual experiments. For both panels **(A,B)**, an asterisk (*) indicates a statistically significant difference with *p* ≤ 0.05. Error bars represent SEM.

To determine the extent to which *C. parapsilosis* is able to modify host cell injury induced by *C. albicans* infection, pIEC damage was measured in the presence and absence of *C. parapsilosis*. Incubation of a mix of *C. albicans* and *C. parapsilosis* cultures (1:2 ratio) with pIECs caused significantly less damage as compared to infection with *C. albicans* alone (Figure 1A). By itself, *C. parapsilosis* strain 4175 does not damage pIECs (Figure 1A), consistent with the results of our previous study (14). *C. parapsilosis* had the same effect of decreasing pIEC damage caused by two different *C. albicans* strains (Figure 1A), a common laboratory strain (SC5314) and a clinical isolate (A022b), both previously shown to significantly damage pIECs (14).

During incubation of pIECs with *C. albicans*, yeast cells form elongated hyphae that start to penetrate the host cells after ~3 h; *C. parapsilosis* cells, which do not form hyphae, exhibit minimal invasion of pIECs (14). In a mixed infection of pIECs using *C. parapsilosis* and *C. albicans*, we found a reduction in the number of *C. albicans* hyphae able to penetrate pIECs as compared to infection with *C. albicans* alone (Figure 1B). The efficiency of *C. albicans* hyphal morphogenesis in the presence or absence of *C. parapsilosis* did not differ, thus the reduction in invasion was not due to differences in hyphal formation by *C. albicans* (data not shown). We also observed that *C. parapsilosis* cells tended to localize along the surface of *C. albicans* hyphae, raising the possibility that physical interactions between the fungal species were inhibiting *C. albicans* from interacting with and injuring host cells.

### *C. parapsilosis* Cell-Free Culture Fraction Reduces *C. albicans*-Induced pIEC Damage

**Figure 2:**
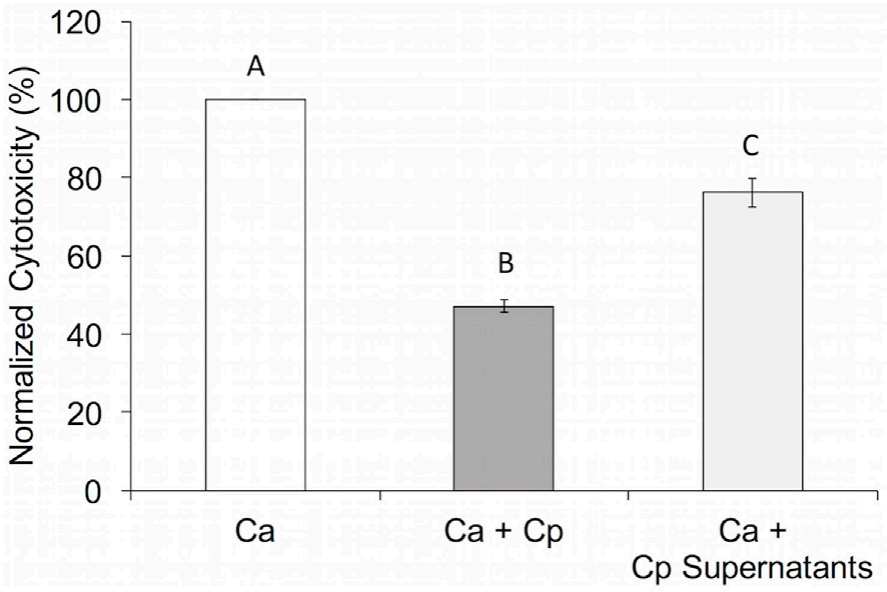
***Candida parapsilosis* cell-free culture fraction reduces *Candida albicans*-induced premature intestinal epithelial cell damage**. Cell damage (lactate dehydrogenase amount) is plotted as a percentage of that caused by *C. albicans* (Ca SC5314) alone (“normalized cytotoxicity”). Data shown are the average of three individual experiments. Letters are used to indicate statistical significance, where letters differ, *p* < 0.05. Error bars represent SEM.

To determine if the *C. parapsilosis* activity that reduces pIEC damage and/or invasion by *C. albicans* is contained in the cell-free culture fraction, *C. parapsilosis* culture supernatants were used to resuspend *C. albicans* cells prior to infection of pIECs. Supernatants were harvested from *C. parapsilosis* cultures grown to the same density (2 × 10^6^ cells/mL) as those used in the invasion and damage assays (Figure 1) to ensure consistent quantities of supernatant components between experiments. For pIEC invasion by *C. albicans* hyphae, addition of *C. parapsilosis* supernatants had no apparent effect, either in reducing or enhancing invasion (data not shown). For pIEC damage, *C. albicans* cells resuspended in *C. parapsilosis* supernatants had significantly reduced ability to damage pIECs, although not as much as when *C. parapsilosis* cells were also present (~20% reduction for supernatants alone; ~50% reduction for supernatants along with cells) (Figure 2). Control *C. albicans* cells that were resuspended in conditioned medium (incubated overnight at 37°C) that was not exposed to *C. parapsilosis* were able to damage pIECs to the same extent as observed previously for unconditioned media (data not shown). These results suggest that *C. parapsilosis* cells secrete a factor that interferes with the ability of *C. albicans* to damage pIECs and that *C. parapsilosis* cells themselves also contribute to reducing damage to pIECs. Of note, *C. parapsilosis* supernatants had no effect on *C. albicans* growth or ability to form hyphae (data not shown). Thus, its function in reducing *C. albicans* damage of pIECs appears to occur independently of these two virulence factors.

### *C. parapsilosis* Reduces the Ability of *C. albicans* to Adhere to pIECs

Fungal adhesion to host cells is thought to be an early step in the pathogenesis of *C. albicans* infections (30, 31). To determine how the *C. parapsilosis* cell-free fraction affects *C. albicans* adhesion to pIECs, we again used supernatants harvested from *C. parapsilosis* cultures as described above. Addition of *C. parapsilosis* supernatants caused an ~15% reduction in the number of *C. albicans* cells able to adhere to pIECs (*p* < 0.05). Thus, the decreased pIEC damage from addition of *C. parapsilosis* supernatants correlates with reduced *C. albicans* adhesion to pIECs.

**Figure 3:**
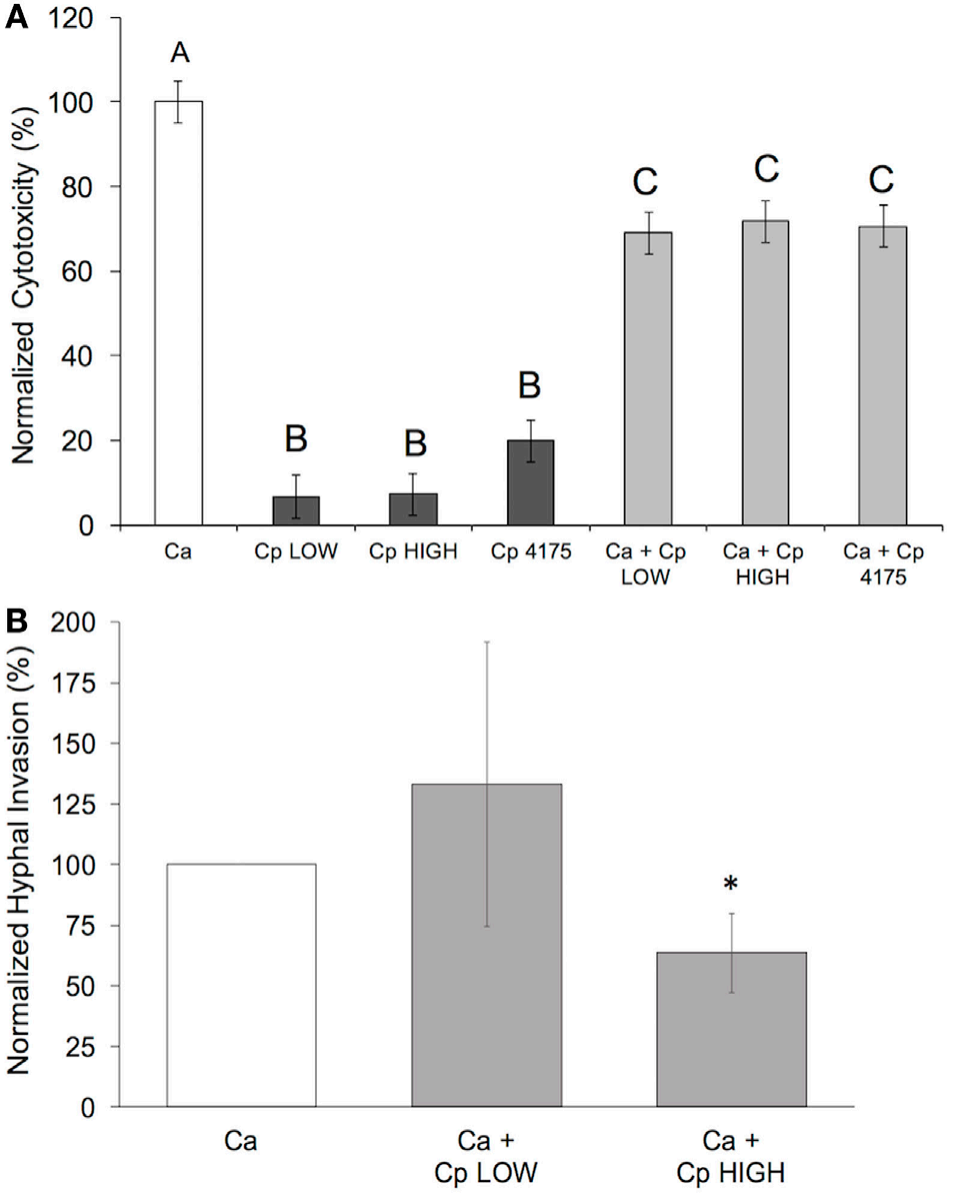
**Adhesiveness of *Candida parapsilosis* strains correlates with ability to reduce *Candida albicans* invasion, but not damage, of pIECs**. **(A)** Cell damage (lactate dehydrogenase amount) is plotted as a percentage of that caused by *C. albicans* (Ca SC5314) alone (“normalized cytotoxicity”). Data shown are the average of three individual experiments. Letters are used to indicate statistical significance, where letters differ, *p* < 0.05. Error bars represent SEM. **(B)** *C. albicans* hyphal invasion in the presence of *C. parapsilosis* (Cp) strains is plotted as a percentage of that observed for *C. albicans* (Ca SC5314) alone. Data shown are the average of three individual experiments. An asterisk (*) indicates a statistically significant difference with *p* ≤ 0.05. Error bars represent SEM.

**Figure 4:**
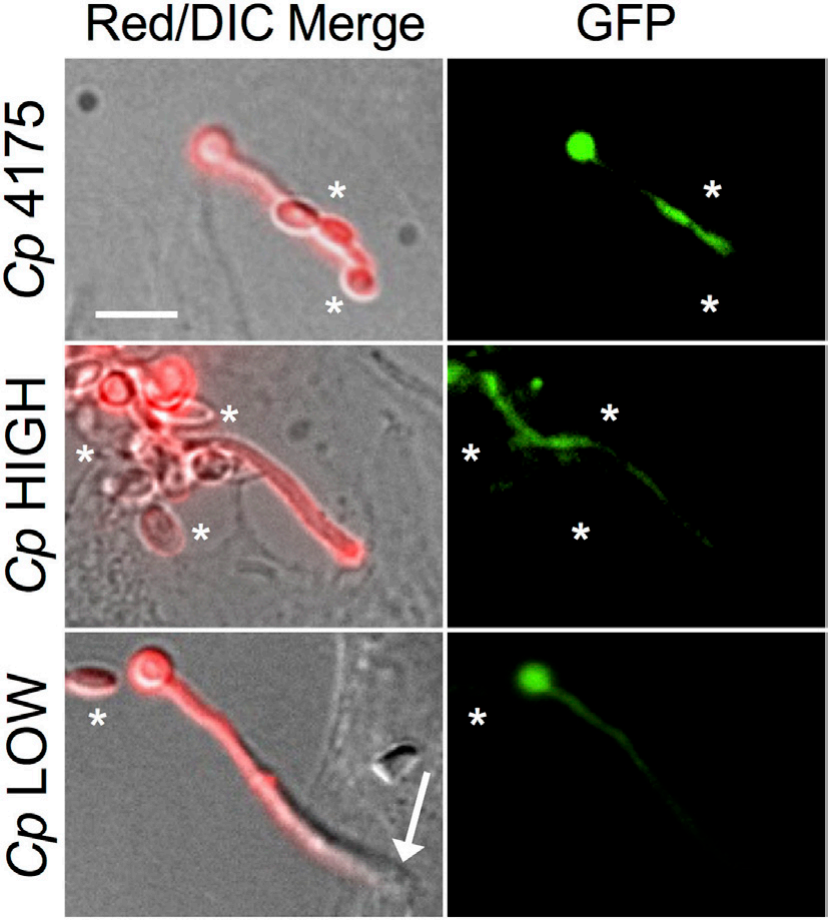
***Candida albicans* and *Candida parapsilosis* interactions on premature intestinal epithelial cells (pIECs)**. Representative photomicrographs of *C. albicans* (*Ca* GFP) coincubated with *C. parapsilosis* (*Cp*) strains on pIECs. A *C. albicans* strain expressing GFP was used to differentiate between *C. albicans* (red and green fluorescence) and *C. parapsilosis* (red fluorescence, no green fluorescence, see asterisks in GFP panels) yeast cells. Alexa 568 conjugated to phalloidin was used to stain fungal cells with red fluoroscence, as previously described (14). A penetrating *C. albicans* hypha lacks red fluorescent signal (*Cp* LOW, Red/DIC Merge panel, arrow). Non-penetrating *Candida* cells exhibit red fluorescent signal. *Cp* 4175 and *Cp* HIGH cells make contacts with *C. albicans* hyphae (top and center merge panels, asterisks), while *Cp* LOW cells do not (bottom merge panel, asterisk). Scale bar, 10 μm.

Most adhesion assays, including the one employed by us as described above, utilize a cell staining method to quantify yeast cells spectrophotometrically. These methods cannot distinguish cells of different yeast species from each other, thus we are not able to determine the effect of *C. parapsilosis* cells on the adhesion of *C. albicans* to pIECs. As an alternative, we explored the extent to which adhesiveness of *C. parapsilosis* cells contributes to reducing pIEC damage caused by *C. albicans* by comparing two *C. parapsilosis* strains that differ with respect to adhesion to human FaDu epithelial cells (22). By themselves, both of these *C. parapsilosis* strains caused a similar, low amount of damage to pIECs that did not differ from that observed with *C. parapsilosis* strain 4175 (Figure 3A). Both strains were able to reduce *C. albicans*-induced pIEC damage (similar to strain 4175) and did not differ with respect to their specific activities. Thus, adhesiveness to FaDu cells does not correlate with differences in the ability of *C. parapsilosis* to inhibit pIEC damage by *C. albicans*. By contrast, pIEC invasion by *C. albicans* hyphae was reduced by coincubation with *C. parapsilosis* strain 4175 (Figure 1B) and the FaDu high-adhesion strain (Figure 3B), but not with the low-adhesion strain (Figure 3B). Furthermore, both strain 4175 and the FaDu high-adhesion strain were observed to localize along *C. albicans* hyphae whereas the FaDu low-adhesion strain was not (Figure 4). Thus, adhesiveness of *C. parapsilosis* strains correlates with their abilities to interact with *C. albicans* hyphae and to reduce *C. albicans* invasion of pIECs.

### *C. parapsilosis* Supernatants Protect Zebrafish from Infection by *C. albicans*

**Figure 5:**
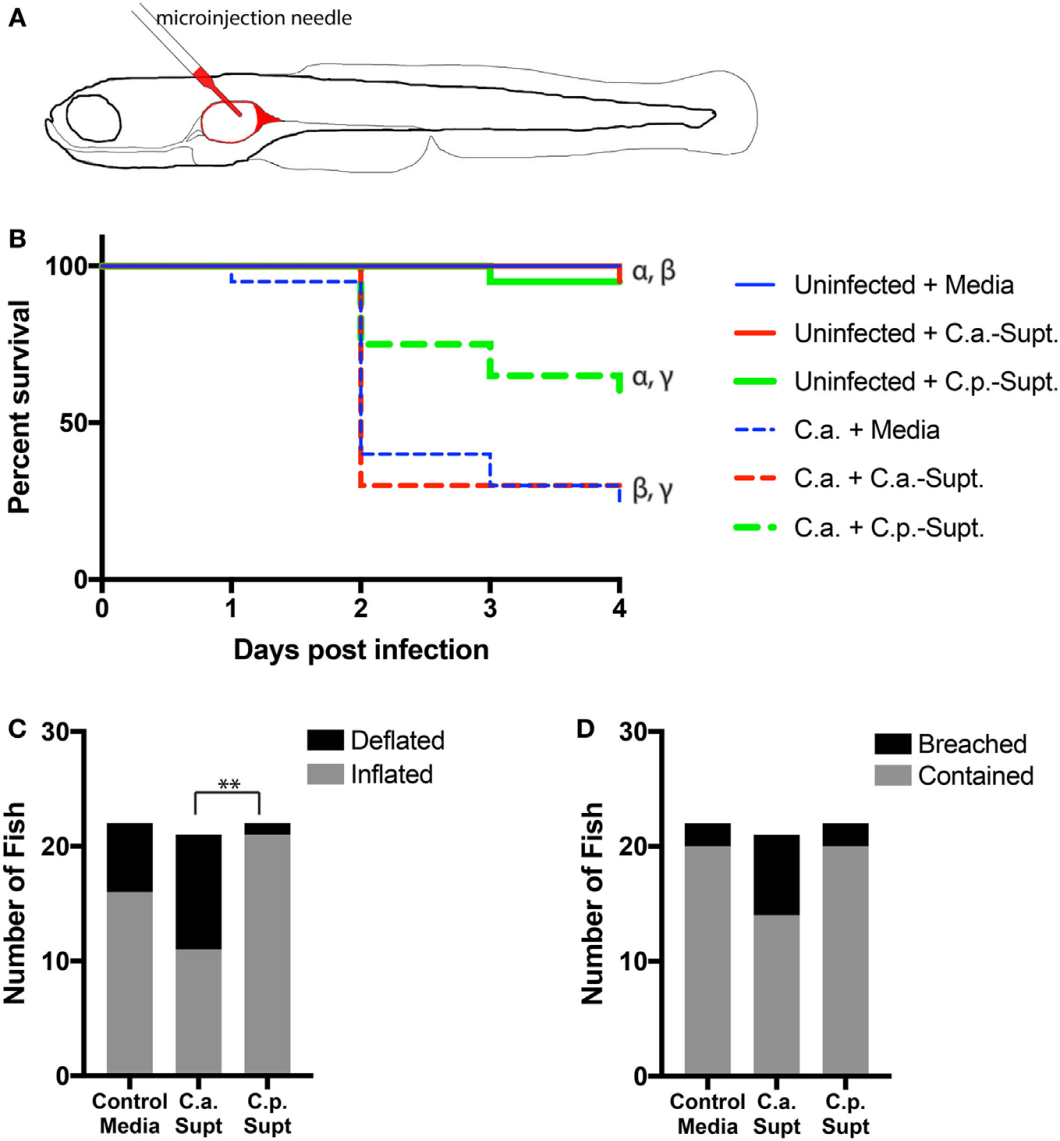
***Candida parapsilosis* cell-free culture fraction protects zebrafish from infection by *Candida albicans***. **(A)** Schematic of infection model. The larval zebrafish swimbladder offers a transparent vertebrate mucosal infection model that is amenable to non-invasive imaging of both the host and the pathogen. **(B–D)** Zebrafish at 4 days post-fertilization with inflated swimbladders were infected in their swimbladders by glass needle injection with *C. albicans* yeast cells (C.a.) suspended in control (H4) media or in supernatants from *C. parapsilosis* (C.p. Supt) or *C. albicans* (C.a. Supt.) cultures. **(B)** Relative survival of fish infected with *C. albicans* with or without *Candida* supernatants. All *C. albicans*-infected fish cohorts are significantly different from their respective controls. *C. parapsilosis* supernatants significantly reduce the mortality of a *C. albicans* infection (denoted by γ). Matching Greek letters label individual comparisons: α, *p* < 0.01; β, *p* < 0.0001; γ, *p* < 0.05. Survival data are pooled from two independent experiments, *n* = 20 per group. All pair-wise comparisons were made with the Mantel–Cox test. **(C,D)** Fish from the experiment in **(B)** were viewed by fluorescence microscopy at 24 h post-infection and scored for two indicators of infection, swimbladder deflation [**(C)** **p < 0.01] and breaching of epithelial barrier **(D)**. Examples of these phenotypes are shown in Figure S1 in Supplementary Material. Data were pooled from two independent experiments and analyzed by Fisher’s exact test with Bonferroni correction (Control media, *n* = 22, *C.a*. supernatant, *n* = 21, *C.p*. supernatant, *n* = 22).

The zebrafish swimbladder is a mucosal organ that is used for buoyancy and is both physiologically and ontologically most closely related to the mammalian lung (Figure 5A) (32–34). To test the protective ability of *C. parapsilosis in vivo*, zebrafish were infected in the swimbladder with *C. albicans* yeast suspended in *C. parapsilosis* supernatant, *C. albicans* supernatant, or unconditioned medium. This infection model recapitulates a number of aspects of *in vitro C. albicans*–epithelial interactions, permits mucosal infection in the context of a vertebrate immune system and allows for high inoculum doses that cause infection without immunosuppression (28, 29). Therefore, this optically transparent disease model is more complex than *in vitro* challenge of epithelial cells but is not as complex as the mouse intestinal colonization model, which is reliant on antibiotic treatment for colonization and requires both physical disturbance and immunosuppression to yield lethal infection (35). Cell-free fractions of *C. albicans* and *C. parapsilosis* cultures, as well as medium alone, had no effect on zebrafish viability. When *C. albicans* was suspended in *C. parapsilosis* supernatant, mortality was significantly reduced (Figure 5B) as compared to *C. albicans* suspended in its own supernatant or fresh unconditioned medium. The effect of *C. parapsilosis* cells along with cell-free fractions on zebrafish viability could not be tested due to the number of yeast in the mixed inoculum being beyond the physical constraints of microinjection into the zebrafish swimbladder.

Deflation of the swimbladder and breaching of the swimbladder epithelium by hyphae are visual hallmarks of *C. albicans* infection in zebrafish (29). Nearly half of the fish infected with *C. albicans* suspended in *C. albicans* supernatant experienced swimbladder deflation at 24 hpi. By contrast, significantly fewer fish had deflated swimbladders when *C. albicans* was suspended in *C. parapsilosis* supernatants (Figure 5C). In addition, there was a trend toward more breaching of the swimbladder epithelium by *C. albicans* hyphae with addition of *C. albicans* supernatant than for *C. parapsilosis* supernatant or control media, although this difference did not reach statistical significance (Figure 5D). Together, these results indicate that *C. parapsilosis* supernatants protect zebrafish from the effects of *C. albicans* infection.

## Discussion

**Figure 6:**
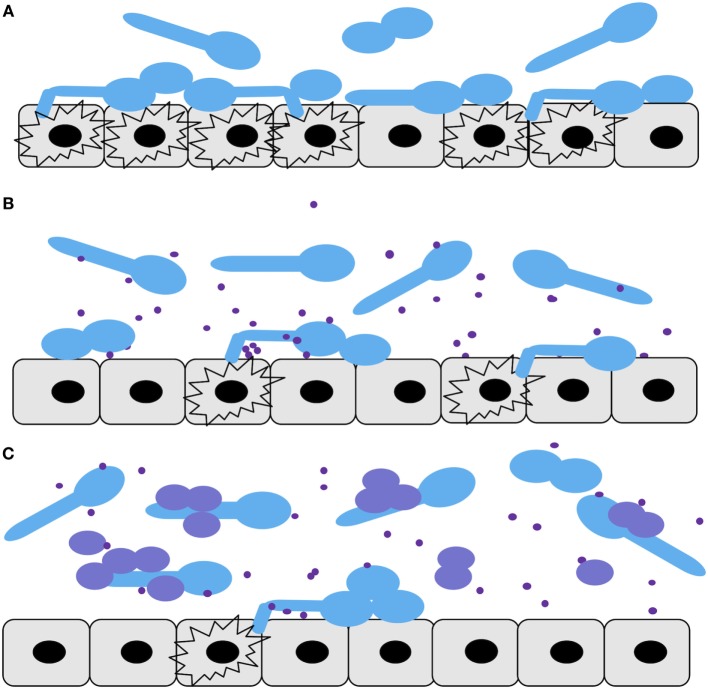
**Diagram summarizing the *Candida*–premature intestinal epithelial cell (pIEC) interactions described in this study**. Infection models: **(A)** *Candida albicans* alone, **(B)** *C. albicans* along with *Candida parapsilosis* cell-free culture fraction, **(C)** mix of *C. albicans* and *C. parapsilosis* cultures. For all panels: *C. albicans* yeast and hyphal cells, light blue; *C. parapsilosis* cells, purple; *C. parapsilosis* cell-free culture factors, purple dots; pIECs, gray; damaged pIECs, black explosion outlines.

Three epithelial cell infection models were compared in this study (Figure 6): *C. albicans* alone (Figure 6A), *C. albicans* with the addition *C. parapsilosis* cell-free fractions (supernatants) (Figure 6B), and a mix of *C. albicans* and *C. parapsilosis* cultures (Figure 6C). Comparison of *C. parapsilosis* cellular versus cell-free culture fractions allowed us to study the relative contributions of each and gain mechanistic insight with respect to pathogenic interactions of *C. albicans* with pIECs. The amount of *Candida* cells that we used for infection of pIECs (~10^5^ cells/mL) is within the range of colonization levels that have been reported in a few studies of fecal fungal colonization of infants and children (~10^1^–10^8^ cells/mL), with the higher levels being more typical for subjects with concurrent diarrhea (5, 36, 37). Thus, we think that our infection models are within a physiologically relevant range. Although infection with two fungal pathogens may be expected to cause more host cell damage, we found that *C. parapsilosis* reduces pathogenic interactions of *C. albicans* with host cells. This is consistent with the idea that pathogens can behave as commensals if they coexist with other microbes that are capable of modulating their virulence functions. Conversely, it is also possible that pathogens could exhibit heightened virulence functions depending on the neighboring microflora. It is notable that the zebrafish swimbladder is not sterile, so the conservation of a protective effect in this mucosal model suggests that this work has relevance *in vivo*. As is true for all studies with infection models, the effects on virulence that we observe must be tested and corroborated in further animal and, eventually, human studies before being brought to the clinic.

*Candida parapsilosis* supernatants alone were able to reduce *C. albicans* adhesion to, and damage of, pIECS (Figure 6B), and occurred independent of effects on *C. albicans* growth or hyphal formation. It seems reasonable that *C. albicans* would need to establish and maintain close contact with pIECs in order to damage them *via* physical penetration and/or *via* targeted delivery of membrane-degrading enzymes such as secreted aspartyl proteinases (SAPs) or candidalysin (38–40). The result that *C. parapsilosis* supernatants reduce pIEC damage by *C. albicans*, then, may be related to their ability to reduce *C. albicans* contacts with host cells, potentially *via* competitive blockade of hydrophobic contacts or fungal adhesin–host receptor interactions (41). In addition, or alternatively, *C. parapsilosis* supernatants may contain a factor that inhibits *C. albicans* secreted enzymes (e.g., SAPs) that degrade pIEC membrane components or that alter the biology of the pIECs (e.g., downregulate a receptor) such that they are less susceptible to adhesion and damage. Addition of *C. parapsilosis* cells along with their supernatants had a greater protective effect against *C. albicans*-induced pIEC damage than with the addition of supernatants alone (Figure 6C). The result that there is an additive effect of *C. parapsilosis* cell and cell-free fractions suggests that each of them is acting *via* different inhibition mechanisms.

It seems likely that fungal-host cell contact would also be important for *C. albicans* to successfully invade pIECs. In support of this mechanistic step, when *C. parapsilosis* cells plus supernatants were coincubated with *C. albicans*, only the *C. parapsilosis* strains that were seen to localize along *C. albicans* hyphae were able to reduce *C. albicans* invasion of pIECs. *C. parapsilosis* supernatants by themselves also reduce the ability of *C. albicans* cells to adhere to pIECs; however, they appeared to have no effect on *C. albicans* invasion of pIECs. Importantly, the invasion assay employed here only analyzes the invasion efficiency of those fungal cells that are already in contact with pIECs (in the same microscopic plane of view). No conclusion can be drawn, then, about the connection between adhesion and invasion using this particular assay. We think it likely that, in the presence of *C. parapsilosis* supernatant, more *C. albicans* cells never made contact with pIECs and were washed away during the invasion assay. Consistent with a protective effect of *C. parapsilosis* supernatants in inhibiting *C. albicans* invasion of epithelia, although not statistically significant, cell-free fractions tended to reduce the ability of *C. albicans* to penetrate the epithelial lining of zebrafish swimbladders (Figure 5D). Thus, our collective data suggest that *C. albicans* invasion and injury of pIECs can be prevented by both *C. parapsilosis* cell and cell-free fractions (Figures 6B,C), either partially or completely, by inhibiting *C. albicans* adhesion to the host cell.

Two mechanisms have been described for *C. albicans* invasion of adult intestinal epithelial cells: active, physical penetration by hyphae, and endocytosis induced by protein–protein interactions (12). For pIECs, our laboratory has observed that the primary mechanism of invasion is penetration by hyphae; only live, actively elongating hyphae are capable of penetrating pIECs (Sara Gonia and Cheryl A. Gale, unpublished data). The identity of the specific factors and processes that mediate hyphal invasion of pIECs remain unknown, but fungal adhesion, physical forces, and targeted secretion of host membrane-degrading enzymes (e.g., proteinases, lipases, candidalysin) from the hyphal tip are likely to be involved as these features have been shown to facilitate invasion of other human cell lines (31, 38).

Cell-free fractions from the yeast *Saccharomyces boulardii* also appear to have a protective effect with respect to pathogenic interactions of *C. albicans* with human intestinal epithelial cells. *S. boulardii* supernatants reduce adhesion of *C. albicans* to adult Caco-2 cells and this is associated with decreased IL-8 cytokine release by enterocytes (25). *Saccharomyces* species are typically regarded as non-pathogenic for the majority of individuals, except in rare cases of severe immunosuppression (42). *C. parapsilosis*, on the other hand, is regarded as a pathogen, particularly in infants (1, 43). Thus, its role in protection against *C. albicans*-induced mortality in zebrafish, and injury and invasion of epithelia *in vitro*, seems somewhat counter-intuitive. Of note, although the same *C. parapsilosis* clone has been isolated from both the intestinal tract and blood of premature infants with fungal sepsis (4), *C. parapsilosis* consistently causes minimal to no damage of pIECs [Figures 1 and 5; (14)]. These observations suggest that *C. parapsilosis* gains entry to the host *via* alternative sites [e.g., intravascular catheters and endothelium (44)] and support the idea that pathogenesis mechanisms employed by *Candida* species are host site- and species-specific (12).

In summary, *C. parapsilosis* reduces *C. albicans*-induced injury and invasion of pIECs and these effects are at least partially due to the ability of both secreted and cellular *C. parapsilosis* factors to reduce *C. albicans* physical interactions with host cells. Further studies are needed to understand the molecules on the surface of *C. parapsilosis* as well as the secreted factors that inhibit *C. albicans* adhesion to, and injury of, pIECs. Potential cell-surface candidates include homologs of *C. albicans* Als proteins and other adhesins (45, 46). For secreted factors, identification of molecules in supernatant fractions that confer inhibitory activities will provide additional mechanistic insight. Ultimately, a more complete understanding of microbe–microbe and host–microbe interactions that are associated with protection from disease may lead to the development of therapeutic strategies that promote intestinal health in at-risk patient populations.

## Author Contributions

SG designed and performed experiments, analyzed data, and wrote the manuscript draft. LA designed and performed experiments, analyzed data, assisted in manuscript writing, and revised the manuscript. MS, MA, and EF performed experiments, analyzed data, and revised the manuscript. JB assisted in development of the project, data analysis, and writing and revision of the manuscript. RW assisted in development of the project, designing of experimental approach, data analysis, and manuscript writing, and revised the manuscript. CG conceived the project, assisted in experimental design, data analysis, and writing of the manuscript, and revised the manuscript.

## Conflict of Interest Statement

The authors declare that the research was conducted in the absence of any commercial or financial relationships that could be construed as a potential conflict of interest.

## Supplementary Material

Video S1 **Animated Z-stack of Figure S1A in Supplementary Material**. Z-stack of a representative fish with partial swimbladder deflation and epithelial invasion. In fish like this representative, the swimbladder retains its air bubble (black oval). *Candida albicans* hyphae (purple) fill the distended space behind the air bubble and grow along the epithelial lining of the swimbladder. Filaments can be seen penetrating the epithelial barrier and invading the tissue under and behind the swimbladder. The animation begins near the midline of the fish and moves outward to the skin. Interslice interval = 5 μm. Scale bar = 150 μm.

Video S2 **Animated Z-stack of Figure S1B in Supplementary Material**. Z-stack of a representative fish with complete swimbladder deflation and extensive invasion of epithelium. In fish like this representative, the swimbladder becomes completely deflated during infection. Here, many *Candida albicans* hyphae have breached the swimbladder epithelium, have penetrated the tissues surrounding the swimbladder, and are even growing through the skin. The animation begins near the midline of the fish and moves outward to the skin. Interslice interval = 5 μm. Scale bar = 150 μm.
