# Supplementary figures and images for: Candida parapsilosis Protects Premature Intestinal Epithelial Cells from Invasion and Damage by Candida albicans
Source: Front Pediatr. 2017 Mar 22;5:54. doi: 10.3389/fped.2017.00054 (PMC5360698; doi:10.3389/fped.2017.00054)

(A)

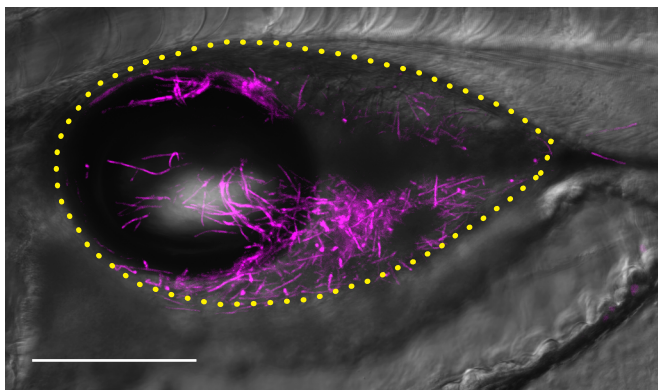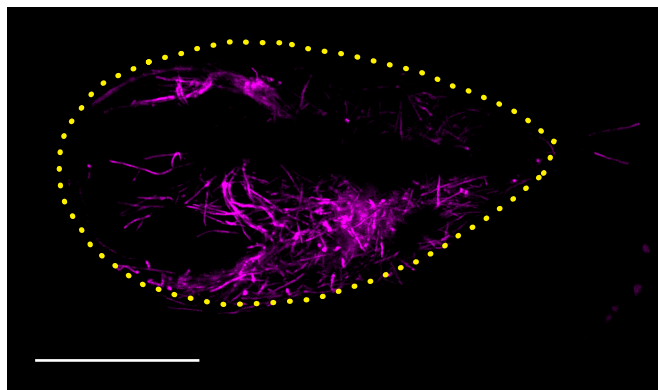

(B)

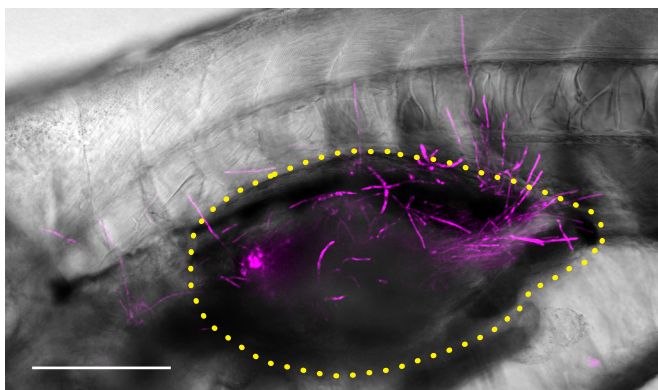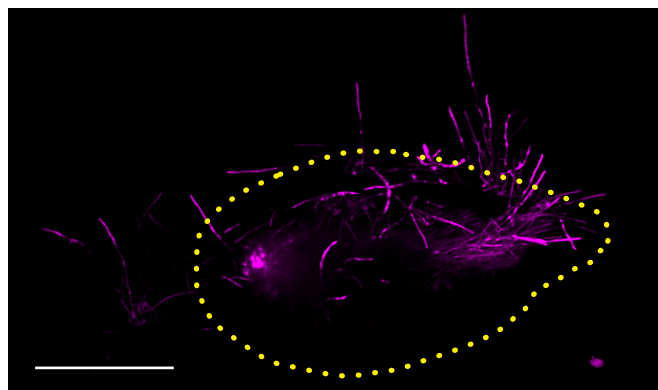

Supplement: Supplementary file 1 [file Image_1.PDF]
